# 

**Published:** 1907-06

**Authors:** 


*The following donations have been received since the publication of the List in March.*

*May* 31st, 1907.

| | |
|---|---|
| J. Paul Bush, C.M.G. (1) .. .. .. .. | 3 volumes. |
| L. M. Griffiths (2) .. .. .. .. .. | 10 ,, |
| R. Shingleton Smith, M.D. (3) .. .. .. | 2 ,, |
| William Warren Potter, M.D. (4) .. .. .. | 4 ,, |

### SIXTY-FOURTH LIST OF BOOKS.

The titles of books mentioned in previous lists are not repeated.

The figures in brackets refer to the figures after the names of the donors, and show by whom the volumes were presented. The books to which no such figures are attached have either been bought from the Library Fund or received through the *Journal*.

**Adami, J. G.** .. *Inflammation* .. .. .. .. .. .. .. .. .. .. 1907

**Allbutt and H. D. Rolleston, T. C.** *A System of Medicine.* Vol. II., Part 2 1907

**Bland-Sutton, J.** *Gall-Stones and Diseases of the Bile-Ducts* .. .. 1907

**Buchanan, A. M.** *Manual of Anatomy.* Vol. II. .. .. .. .. .. 1907

*Chlorine, The Disinfectant Value of* .. .. .. .. .. .. .. .. .. [N.D.]

**Cocks, W. P.** .. *Treatise on Operative Surgery* .. .. .. .. .. (2) 1837

**Cripps, H.** .. *On Diseases of the Rectum and Anus* .. 3rd Ed. 1907

„ .. *Cancer of the Rectum* .. .. .. .. .. .. .. .. 1907

**Crowley, R. H.** *The Need, Objects, and Method of the Medical Inspection of Primary Schools* .. .. .. .. .. .. 1907

**Deanesly, E.** .. *Modern Methods of Diagnosis in Urinary Surgery* 1907

*Encyclopedia and Dictionary of Medicine and Surgery, Green's* Vol. IV. [1907]

**Feindel, H. Meige and E.** *Tics and their Treatment* (Trans. and Ed. by S. A. K. Wilson) .. .. .. .. .. .. .. 1907

**Gadd, H. W.** .. *A Synopsis of the British Pharmacopœia* (1898) 6th Ed. 1907

**Hare, H. A.** .. *Text-Book of the Practice of Medicine* .. 2nd Ed. 1907

**Herman, G. E.** *Diseases of Women* .. .. .. .. .. [3rd Ed.] 1907

„ .. *First Lines in Midwifery* .. .. .. New Ed. 1907

**Hope, Mrs.** .. *Memoir of James Hope, M.D.* (Ed. by K. Grant) (2) 2nd Ed. 1843

**Lange, F.** .. *Degeneration in Families* (Trans. by C. C. Sonne) 1907

**Latham, A.** .. *Pulmonary Consumption* .. .. .. .. .. 3rd Ed. 1907

**Lettsom, J. C.** *Hints designed to promote Beneficence, &c.* .. (2) Vols. II., III. 1801

**Lister, Lord** .. *The Third Huxley Lecture* .. .. .. .. .. (1) 1907

**Lloyd, W.** .. *Hay-Fever, Hay-Asthma* .. .. .. .. .. .. .. 1907

**McKisack, H. L.** *A Dictionary of Medical Diagnosis* .. .. .. .. .. 1907

**Meige and E. Feindel, H.** *Tics and their Treatment* (Trans. and Ed. by S. A. K. Wilson) .. .. .. .. .. .. .. 1907

**Page, F. J. M.** *Elements of Physics* .. .. .. .. .. .. .. .. .. 1907

*Palmer, "The Times" Report of the Trial of William* .. .. .. (2) 1856

**Rabagliati, A.** *The Functions of Food in the Body* .. .. .. .. 1907

**Ramsay, A. M.** *Eye Injuries* .. .. .. .. .. .. .. .. .. .. .. 1907

**Rolleston, T. C. Allbutt and H. D.** *A System of Medicine* Vol. II., Pt. 2 1907

**Von Noorden, C.** *Metabolism and Practical Medicine* (Ed. by I. Walker Hall). Vols. I., II. .. .. .. .. .. 1907

**Walker, J. W. T.** *The Renal Function in its Relation to Surgery* .. 1907

**Wells, J. W.** .. *The Influence of Cod-liver Oil on Tuberculosis* .. 1907

### TRANSACTIONS, REPORTS, JOURNALS, &c.

American Association of Obstetricians and Gynecologists, Transactions of the .. (4) Vols. XII., 1900; XV.—XVII. 1903—1905

American Journal of the Medical Sciences, The .. Vol. CXXXII. 1906

American Medicine .. .. .. .. .. .. .. .. .. .. N.S., Vol. I. 1906

American Society of Tropical Medicine, Papers of the Vol. II. 1905—1907

American Surgical Association, Transactions of the .. Vol. XXIV. 1906

Annales de Dermatologie et de Siphilographie .. .. Tom. VII. 1906

Archives de Neurologie .. .. .. .. .. .. Tom. XXI., XXII. 1906

| | | |
|---|---|---|
| Archives of Pediatrics | Vol. XXIII. | 1906 |
| Australasian Medical Gazette, The | Vol. XXV. | 1906 |
| Bookseller, The | | 1906 |
| Bristol Medico-Chirurgical Journal, The | Vol. XXIV. | 1906 |
| British Journal of Children's Diseases, The | Vol. III. | 1906 |
| British Journal of Dermatology, The | Vol. XVIII. | 1906 |
| Bulletin de l'Académie de Médecine | Tom. LV., LVI. | 1906 |
| Bulletin de l'Académie royale de Belgique | Tom. XX. | 1906 |
| Canadian Practitioner and Review, The | Vol. XXXI. | 1906 |
| College of Physicians of Philadelphia, Transactions of the | Vol. XXVIII. | 1906 |
| Congrès (XV^me.) international de Médecine, 1906 (3) Sect. de Médecine, 2 parts, 1906 ; (1) Chirurgie, 2 parts | | 1906—1907 |
| Contributions from the Department of Neurology, Harvard Medical School | Vol. II. | 1907 |
| Gazette des Hôpitaux de Toulouse | | 1906 |
| Hospital, The | Vol. XL. | 1906 |
| Hospital— | | |
| Johns Hopkins Hospital Reports | Vols. XIII., XIV. | 1906 |
| Indian Medical Gazette | Vol. XLI. | 1906 |
| Johns Hopkins Hospital Bulletin | Vol. XVII. | 1906 |
| Journal of Hygiene, The | Vol. VI. | 1906 |
| Journal of Mental Science, The | Vol. LII. | 1906 |
| Journal of Nervous and Mental Disease, The | Vol. XXXIII. | 1906 |
| Journal of Obstetrics and Gynæcology, The | Vol. X. | 1906 |
| Journal of the American Medical Association, The | Vol. XLVII. | 1906 |
| Journal of the Royal Sanitary Institute | Vol. XXVII. | 1904 |
| Library, The | N.S., Vol. VII. | 1906 |
| Lunacy, 31st Report of the Commissioners in | (2) | 1877 |
| Luzerne County Medical Society, Transactions of the | Vol. XIV. | 1906 |
| Medical Annual, The | | 1907 |
| Medical Record | Vol. LXX. | 1906 |
| Montreal Medical Journal, The | Vol. XXXIV. | [1905] |
| Münchener medizinische Wochenschrift | Bd. II. für | 1906 |
| Northumberland and Durham Medical Journal, The | | 1906 |
| Post-Graduate, The | Vol. XXI. | 1906 |
| Progressive Medicine | Vol. I. | 1907 |
| Revue générale d'Ophtalmologie | Tom. XXV. | 1906 |
| Rhode Island Medical Society, Transactions of the | Vol. VII., Pt. 3 | 1906 |
| Smithsonian Report for 1905 | | 1906 |
| Therapeutic Gazette, The | Vol. XXX. | 1906 |
| Treatment | Vol. X. | 1906 |
| Wiener klinische Wochenschrift | | 1906 |
| Zentralblatt für innere Medicin | | 1906 |

### Local Medical Notes.

**University College, Bristol.**—*Examination Results.*—The following successes have recently been gained:—

CONJOINT BOARD.—*Biology:* B. G. Derry, A. G. T. Fisher, G. H. Piercy. *Anatomy and Physiology:* R. C. Clarke, J. F. H. Morgan. *Medicine:* J. W. J. Willcox.* *Surgery:* A. E. Iles, E. J. Dermott, P. S. Connellan,* C. E. K. Herapath.* *Midwifery:* R. G. Vaughan.

L.D.S.—*Chemistry and Physics:* F. C. Willows. *Mechanical Dentistry and Dental Metallurgy:* G. F. Fawn, W. E. Drummond, I. R. Hudleston, P. J. Burton. *Final Examination:* C. A. Joll.* *Part II. only:* W. H. Ireland.*

L.S.A.—*Medicine (Part I.) and Forensic Medicine:* J. F. McQueen.

*Appointments.*—D. S. Davies, M.D. Lond., has been appointed Examiner in State Medicine for the University of London. C. E. K. Herapath, M.R.C.S., L.R.C.P., has been appointed Casualty Officer at the Bristol Royal Infirmary, Cyril C. Lavington, M.B., B.S. Durh., has been appointed Out-Patient Physician to the Bristol Children's Hospital. Frederick H. Rudge, M.R.C.S., L.R.C.P., has been appointed House Surgeon to the Torbay Hospital, Torquay. E. H. E. Stack, M.B., B.C. Cantab., F.R.C.S. Eng., has been appointed Honorary Assistant Surgeon to the Bristol Eye Dispensary.

**Bristol General Hospital.**—As mentioned in our last issue, a public appeal has been made for £18,000 for this institution for greatly needed improvements. It is announced that £15,550 have already been promised, and it is hoped that the remainder will be obtained shortly.

**Royal United Hospital, Bath.**—The late Dr. Thomas J. Bennett, formerly of Tunbridge Wells, has bequeathed the whole of his estate, subject to certain legacies, amounting to £20,000 to this institution. This sum will be devoted to the augmentation of the permanent endowment of the hospital. This legacy will not interfere with the efforts of the Mayor of Bath, who is trying to extinguish the debt of £6,000 against the hospital.

**Royal Mineral Water Hospital, Bath.**—At the annual meeting of this institution held recently it was stated that 1,185 in-patients had been admitted during 1906, an increase of 24 compared with 1905. The average daily number of occupied beds was 145. An unfavourable balance of £214 on the year's working was reported.

**Winsley Sanatorium, Bath.**—At a recent meeting of the House

* Completes examination.

Committee it was pointed out that certain beds could now be allocated to persons residing within the counties of Somerset, Gloucester and Wilts, on payment of 35s. per week, and persons living outside these districts could be admitted for £2 10s. weekly, provided there were vacant beds not required by residents of the three counties. Application should be made to the Secretary.

**Cardiff Infirmary.**—In the annual report of this institution an appeal has been made for £30,000 in order to accommodate sixty-four additional in-patients, bringing the total number of beds up to 250. The infirmary serves a district comprising a population approaching half a million, and there are constantly between 500 and 600 patients awaiting admission. A promise of £5,000 has already been received provided the remaining £25,000 are subscribed within six months.

**Royal Devon and Exeter Hospital, Exeter.**—At a meeting of the Governors held recently it was reported that the adverse balance against the institution amounted to £1,392, being £866 more than last year. The chairman pleaded for more general support, failing which more funded stock would have to be sold.

**The Workmen's Compensation Act.**—An interesting case, bristling with medical technicalities and details, was heard in a local County Court recently. The applicant was a pork butcher's assistant, aged 26, who sustained an accident on the 2nd October, 1906, straining himself by lifting a heavy lead-lined pickle-tub. His statement was that he felt something had gone wrong, and pain had come in his left shoulder when he got home. He worked on and did not seek medical advice from his club doctor until five weeks after the alleged accident, on a day when he asserted that he fainted while standing at his own fireside doing nothing. During the long period between the accident and this the pains grew gradually worse, and in his own words, his heart "did beat very hard." He was treated for a month in a local hospital, and there seems to have been some doubt as to the diagnosis. The disease, however, was designated subclavian aneurysm. Later on the man brought an action against his master on the grounds that he had sustained an injury in the course of his employment which determined the illness.

Ample scope was given during the inquiry at the Court for the minute discussion of aneurysm in all its bearings. Counsel for the respondent, with his then knowledge of the subject, would have little difficulty in satisfying any medical examining board. Medical witnesses were at the table *vis-à-vis,* and much interest was taken in the case. The point originally raised was that the applicant had strained an artery under the collar bone. What was the disease. Was it aneurysm or something of a less grave nature ?

The Ocean Accident Company sent down one of their medical referees, who with one of the local doctors minutely examined the applicant. Separately at first, and then together, they concluded that the man had no aneurysmal dilatation whatever.

When the case came on for hearing, the diagnosis suddenly veered round from the region of the subclavian artery to the arch of the aorta. Physical signs were adduced—an accentuated second aortic sound and a loud and distinct murmur conducted towards the axilla. There was some pulsation above the clavicle, and the medical witness could feel the artery quite distinctly.

By His Honour the Judge: "And what was your deduction from this?"

Answer: "That the man had strained the arch of the aorta." Later on, in reply to the Counsel for the respondent: "The aneurysm was traumatic, not spontaneous."

Another medical man had examined the patient and signed the disease up as subclavian aneurysm. This was a lever for further cross-examination.

"Would you agree or disagree with this diagnosis?"—"I should not entirely agree."

"Then you disagree on that?"—"I do not entirely disagree. Aneurysms may be of different degrees. I consider it was the beginning of the dilatation which ultimately leads to serious aneurysm."

The Judge: "You say there was aneurysm, but it was so small as not to appreciably make itself apparent?"

Answer: "There was no swelling, but there was undoubtedly slight aneurysm."

By the Counsel: "Was there or was there not an aneurysm? . . . I cannot accept your answer unless you tell me where mere dilatation ends and aneurysm begins."

Answer: "I should say in this sense there was an aneurysm."

Further on by the medical witness: "He is better, he is comparatively well."

The medical opinions on behalf of the respondent were to the effect that the man did not suffer from aneurysm, and had no signs of ever having had such.

The Judge asked whether a small aneurysm in November might not have disappeared in April.

Answer by one witness: "It would have been so small that it could not be diagnosed." Later: "The first and essential step in diagnosing an aneurysm is the presence of a tumour or swelling."

On the question of the man's symptoms the Judge cross-examined one of the medical witnesses:

"Did not the strain of the accident cause all that the medical witness for the applicant had described—Yes or No?—palpitation, for instance, which the man stated he still suffered from six months after the accident."

The Judge seemed very irritated by the medical witness giving a qualifying answer.

"Would not a bicycle ridden too hard cause palpitation—Yes or No?"

The character of the man's pain was scarcely that which one would have expected in an aneurysmal dilatation of the aorta, as he described it, "in his left shoulder shooting up to his ear and down into his heart." The pain was persistent since the accident six months ago.

The decision arrived at by His Honour was easily anticipated. He had to determine whether the applicant's illness was due to an accident, and if so, what was the cause of the accident, and whether it arose in the course of the man's employment. He found that the applicant did meet with an injury from the lifting of the pickle-tub, that he was subsequently ill owing to an undue strain, and he was still suffering from it. Incidentally he remarked that he had had two doctors before him who formed an opposite opinion, but he had to consider what was the means of knowledge they had in forming their opinion. They did not see the man until a long time after the accident, and therefore it was very difficult for them to say what was the cause of an illness that commenced months and months before.

**Cossham Memorial Hospital.**—The late Mr. Handel Cossham, who had represented in Parliament this division of Bristol—the East—for several years and until his death, left in his will his residuary estate for the purpose of establishing a hospital. He had no children, but left a widow, and as he was never considered a wealthy man, it seems most likely that the charitable institution that was present to his mind—generous and beneficent as it was—would at the most be a fair-sized Cottage Hospital.

Owing to various circumstances the estate got into Chancery, and this occasioned a considerable delay in the winding-up. Strange to say, this delay was a fortunate thing, for the Trustees had to manage the collieries and keep them going, and luckily they fell upon very good times in the coal trade, which is always a fluctuating one, and made excellent profits. Not only so, but the collieries were sold at the top of the financial wave; and so, in the end, the grand total of £120,000 was in hand for building and endowment. The site chosen is at the top of Lodge Hill, in the parish of Fishponds, but in close proximity to the populous district of Kingswood; and with the land around the hospital, and two well-made private roads, consists of nearly fifty acres. The ground is high—the highest in the district—and is about 350 feet above sea-level. It commands a most extensive prospect, and especially from the balcony on the central tower. To the north are the Cotswolds, about Wotton-under-Edge; to the east Kelston and Lansdown hills and the Bath districts; to the south the Mendips; and more westerly Bristol itself; whilst to the west are the Severn Valley, the Forest of Dean, and the distant hills of Wales.

The neighbourhood is also a historical one. Years ago it formed part of the king's wood, and almost within a stone's throw of the hospital is a large round brick tower, which is said to represent a hunting-tower in the reign of King John. Below Lodge Hill on the western side runs "The Causeway," an old Roman road to Bath.

The building itself is very imposing, being constructed of the grey stone of the neighbourhood, faced with the best Bath or Corsham stone. The centre block contains the entrance hall, surgeon's and matron's sitting rooms, the reception and board rooms, the surgery, dispensary, and at the back the kitchens and offices. On the western side rises up the tower, already spoken of. This tower has a copper-covered cupola and an illuminated clock (four faced, and furnished with the Westminster chimes). This clock is connected by electric wires with all the time-pieces in the establishment, so that everywhere the time is synchronous. Near this tower, at its base, are the two surgical wards, male and female, and also the great staircase leading up to the first floor, where are also two similar wards, the medical male and female. On the ground floor, to the south of the wards, is the operating theatre. This is one of the handsomest and the best equipped in the country. The floor is laid with white square tiles, slightly toughened on the surface ; the walls are covered with pale green-coloured tiles, highly vitrified and known as "Brit-Opal," made and placed by the Adamant Co., Ltd., Birmingham. Above the cornice line white tiles are used, and carried up into the lantern, which gives light and assists also in the ventilation of the theatre. Where the floor level ends the edges are mitred and decorated with darker tiles, so that here and wherever edges meet, so to speak, everything is rounded off, leaving no edges anywhere for dust or germs to rest. All the tiles are embedded in Portland cement, and secured in place with Birmingham cement. On each side of the theatre is a large radiator, heated with low-pressure steam, and guaranteed by the contractors—Messrs. Bradford—to give a temperature of 75° F. on the coldest winter night. Behind each radiator is an inlet for cold fresh air, and a continuous circulation of air is maintained by two fans in the ceiling. Leading into the theatre are two smaller rooms, all constructed with the same materials, the anæsthetising room and the sterilising one. The sanitary arrangements consist of lavatories, tanks and ample discharge pipes, which terminate in a deep glazed channel which runs the length of one side of the floor.

The water supply consists of pure hot and cold sterilised and ordinary hot and cold water. The supply is introduced by means of copper pipes, including the necessary ties and clips,

½-inch hot and cold water, quarter-turn taps, and wrist-action taps, sprays and douches.

The four wards are all the same size. They are large and spacious, and will furnish ample cubic capacity for the ten adult beds and two cots which are being placed in each one. The floors are laid with cement, and covered with teak blocks. The patients' lockers are also of teak, with brass fittings, and the chairs made of dark-stained wood, all of which will form a good contrast to the best Lawson-Tait bedsteads in white enamel and the bright-coloured bedspreads. In the centre of each ward is a stove, which can be used for extra warming should the radiators alongside the walls be insufficient in cold weather, and near each stove a handsome plate-glass covered table, fitted with drawers at each end.

Leading out of the far end of each ward are lavatories and w.c.'s, furnished with the most approved fittings, and on each floor is a very commodious ward kitchen and larder. Adjoining the wards on each floor there is a day room for patients, the one on the ground floor for females, and the one above for males. This latter one has a covered open-air space and an open balcony, where the men may smoke at times. On each floor are bathrooms for the patients. At the north end of the building are the isolation wards. These are separated from the rest of the building by double doors and an intervening corridor. There are wards on each floor, with separate offices, and nurses' sitting and sleeping rooms. Connected with these wards is an out-door spiral staircase, which can be used in cases of emergency. The same arrangements exist for the other main wards.

In the basement are various offices, but worthy of special note are a dark room for photography, an X-ray chamber, and the *post-mortem* room.

Beyond the main building, westward, is another distinct block, where are the laundry, engine-room, stable, coach-house, and mortuary.

As the capabilities of the hospital in every way are beyond those of the ordinary cottage hospital, the Committee determined to have an honorary working staff and an honorary consulting staff commensurate with these surroundings. The honorary consulting staff is represented by two physicians and two surgeons, and these are respectively Dr. Shingleton Smith and Dr. J. Odery Symes, and Mr. C. A. Morton and Dr. James Swain. The honorary working staff is Dr. Nixon and Dr. Bertram Rogers as the physicians, and Dr. E. Hey Groves and Dr. Stack as surgeons. Mr. A. L. Flemming has been appointed anæsthetist. The house surgeon is Dr. G. C. Mort, of the Victoria University, Manchester, and the matron, Miss Maun, late of the Infirmary, Gloucester.

